# Adaptation of Group A *Streptococcu*s to Human Amniotic Fluid

**DOI:** 10.1371/journal.pone.0009785

**Published:** 2010-03-23

**Authors:** Izabela Sitkiewicz, Nicole M. Green, Nina Guo, Ann M. Bongiovanni, Steven S. Witkin, James M. Musser

**Affiliations:** 1 Center for Molecular and Translational Human Infectious Diseases Research, The Methodist Hospital Research Institute, Houston, Texas, United States of America; 2 Department of Pathology, The Methodist Hospital, Houston, Texas, United States of America; 3 Weill Medical College of Cornell University, New York, New York, United States of America; University of Hyderabad, India

## Abstract

**Background:**

For more than 100 years, group A *Streptococcus* has been identified as a cause of severe and, in many cases, fatal infections of the female urogenital tract. Due to advances in hospital hygiene and the advent of antibiotics, this type of infection has been virtually eradicated. However, within the last three decades there has been an increase in severe intra- and post-partum infections attributed to GAS.

**Methodology:**

We hypothesized that GAS alters its transcriptome to survive in human amniotic fluid (AF) and cause disease. To identify genes that were up or down regulated in response to growth in AF, GAS was grown in human AF or standard laboratory media (THY) and samples for expression microarray analysis were collected during mid-logarithmic, late-logarithmic, and stationary growth phases. Microarray analysis was performed using a custom Affymetrix chip and normalized hybridization values derived from three biological replicates were collected at each growth point. Ratios of AF/THY above a 2-fold change and P-value <0.05 were considered significant.

**Principal Findings:**

The majority of changes in the GAS transcriptome involved down regulation of multiple adhesins and virulence factors and activation of the stress response. We observed significant changes in genes involved in the arginine deiminase pathway and in the nucleotide de novo synthesis pathway.

**Conclusions/Significance:**

Our work provides new insight into how pathogenic bacteria respond to their environment to establish infection and cause disease.

## Introduction

Group A *Streptococcus* (*Streptococcus pyogenes*, GAS) is an exclusively human, Gram-positive pathogen that causes a broad variety of diseases from mild pharyngitis and skin infections to necrotizing fasciitis, streptococcal toxic shock syndrome, and non-suppurative sequelae such as acute rheumatic fever or glumerulonephritis (for a review see [1]). Since the 1980's, GAS has re-emerged as an important cause of severe invasive infections and is estimated to cause approximately 500,000 deaths each year globally despite available antibiotic treatment [2]. In the late 1800's, GAS was identified as a causative factor of puerperal sepsis – a severe invasive infection in post partum women [3]. Due to advances in hospital hygiene, namely physicians washing their hands between deliveries, these types of GAS infections became less frequent. However, in the last three decades there has been a resurgence in GAS infections of the female urogenital tract and vulvovaginatis in pre-pubescent females, and invasive postpartum disease now accounts for approximately 2.2% of invasive GAS diseases [4]. Women with pre-existing throat infections or disrupted mucosal and skin barriers during pregnancy and delivery are particularly susceptible [4].

GAS strains are classified based on the aminoterminal sequence of the M protein, a polymorphic cell surface adhesin and anti-phagocytic factor [1]. Among the various GAS serotypes, serotype M28 strains are a common cause of invasive disease and have an unusual propensity to cause vaginitis and postpartum infections [4]–[6]. Full-genome sequence analysis of an M28 strain provided the first insight into the underlying molecular mechanism for the ability of these strains to cause a disproportionate number of postpartum infections [5]. Green et al. identified a mobile genetic element, named Region of Difference (RD) 2, that was present in all serotype M28 strains analyzed and a subset of other M serotype strains that have been linked epidemiologically to maternal-neonatal infections [5]. Interestingly, RD2 shares extensive similarity with regions in group B *Streptococcus* (*Streptococcus agalactiae*, GBS) [5], [7], which is a common cause of female urogenital tract infections, and genes in groups C and G *Streptococcus* (Sitkiewicz I., Green NM., and Musser JM, unpublished) that can also cause severe invasive infections [8], [9].

As mentioned previously, GAS is capable of causing a wide range of diseases by successfully colonizing a variety of anatomical sites. Transcriptome analyses of GAS grown in blood, saliva, epithelial cells, and polymorphonuclear leukocytes (PMNs) have revealed that GAS is extremely adaptable and modifies its transcriptome based on environmental signals [10]–[13]. To establish infection in the female urogenital tract, GAS must survive and replicate in a bacteriostatic host environment. The ability of GBS to persist and replicate in human amniotic fluid (AF) [14] is an important contributing factor to its ability to cause severe pre- and postpartum infections, despite AF having antibiotic properties towards other bacterial species [15]. Because M28 GAS strains contain the RD2 element and cause a disproportionate number of postpartum infections, we hypothesized that serotype M28 GAS would be able to survive and persist in human AF, similarly to GBS. To initially characterize the interaction of pathogenic GAS within this specific host niche, we used an ex vivo strategy to analyze the global transcriptional response of GAS grown in human AF compared to GAS grown under standard laboratory conditions.

## Materials and Methods

### Bacterial strains and routine growth

GAS strains MGAS6180 (serotype M28, RD2+) [5], and MGAS 5005 (serotype M1, RD2-) [16] were grown in Todd Hewitt medium with 0.5% yeast extract or on TSA II plates supplemented with 5% sheep blood (BD Diagnostics) at 37°C in 5% CO_2_.

### Growth of bacteria in AF

Human (AF) was collected from pregnant women seen at The Methodist Hospital, Houston, Texas, or Weill Medical College of Cornell University in New York City. Samples were collected in accordance with an exempt human subjects protocol approved by the Institution Review Board of each institution. The study involved collection of existing diagnostic specimens routinely collected during clinical procedures as amniocenteses and would have been otherwise discarded. Specimens were stripped of all identifiers and processed in a manner that subjects cannot be directly or indirectly identified.

AF samples were tested to determine if they supported bacterial growth. GAS cells were grown overnight in THY, washed twice in sterile PBS, and re-suspended in PBS to 100×. Ten µl of the 100× bacteria suspension were diluted further in PBS and were used to inoculate each 250 µl sample of heat inactivated (95°C, 5 min) AF, resulting in a final average inoculum of ∼10^4^ CFU/ml. Samples were incubated at 37°C with 5% CO_2_ for 24 h. To avoid THY carryover that might bias the growth results, bacteria were diluted 1∶50 or 1∶25 into a fresh aliquot of AF after the first 24 h. Aliquots were removed, serially diluted, and plated on TSA II plates (BD Diagnostics) every hour for the first 12 h and every 12 h thereafter for CFU enumeration. AF samples that supported growth of GAS were pooled and used for the microarray analysis. Samples were collected from three pooled AF cultures (biological replicates) after 3.5, 5, and 9 hours of growth in AF, which corresponds to bacteria in mid log (ML) growth phase, late log (LL, time point corresponding with the transition from log to stationary phase) and stationary (S) phase, respectively. Bacteria grown in THY laboratory medium were collected in ML, LL and S phase from three independent cultures (biological replicates).

### RNA isolation

The bacteria used for RNA isolation were mixed with 2 volumes of RNA Protect reagent (Qiagen) and cells were collected by centrifugation and stored at −80°C until processing. RNA from GAS was isolated as described previously [17]. All samples were processed at the same time to minimize experimental error. Reverse transcription, cDNA fragmentation, and labeling for all samples were performed as described previously [17].

### Microarray analysis

Microarray analysis was performed using a custom-made Affymetrix chip [18] that contained 1929 redundant probes representing the core GAS chromosome and 289 redundant probes unique for MGAS6180. A total of 1765 MGAS6180 genes were represented on the array. Chip hybridization and data acquisition and processing were performed as described [19]. Samples used for microarray analysis were collected from cultures in THY and AF at mid logarithmic (ML), late logarithmic (LL) and stationary (S) growth phases. The average expression values for each transcript in AF in each growth phase was divided by the average expression in THY to generate AF/THY ratios and degree of changes. Genes that had a two-fold change of expression or greater in AF and were statistically significant (P<0.05) were included in the analysis.

The data is deposited in MIAME-compliant GEO database under accession number GSE19985.

## Results and Discussion

### Characterization of GAS growth in AF

Human AF is a nutritionally poor environment. AF is composed primarily of water and low amounts of sugars and proteins, levels of which decrease as the pregnancy nears term [20]. Recent detailed compositional analysis of AF revealed the presence of multiple proteins (serum albumin, transferrin, α-I-Antitripsin, α-fetoprotein, calpain 6, pinin, type XIII collagen, immunoglobulins) [21], glucose, fructose, lipids, hormones (estrogen and progesterone), and epithelial cells. Sources of AF are predominantly fetal urine (∼900 ml per day influx at term), tracheal fluid, fetal lung fluid, and water and solvents transferred between AF and fetal blood in placenta [22].

GAS rarely causes septic abortions [4]; however, after disruption of membranes, release of AF can change the environment of the female genital tract by increasing pH from acidic to neutral/slightly alkaline and affect balance of natural bacterial vaginal flora. GAS grows better in neutral than acidic pH [23], therefore pH change can promote growth of colonizing pathogens and postpartum infection. Because GAS is able to cause postpartum infections suggests that it is able to survive in an environment containing AF.

Individual specimens of AF collected from women at various stages of pregnancy can vary in their bacteriostatic properties, primarily due to the activity of lysozyme, immunoglobins, and β−lysin [24]–[26]. In general, samples from early stage pregnancies are less bacteriostatic than samples from late stage pregnancies [27]. In addition, bacteriostatic properties of AF also depend on the presence of meconium and iron availability [28]. Often the presence of meconium in AF is used to predict the likelihood of the mother developing a postpartum or intrapartum bacterial infection [29].

Therefore we wanted to determine if GAS can survive in bacteriostatic AF and if so, what transcriptional changes it undergoes to achieve this.

**Figure 1:**
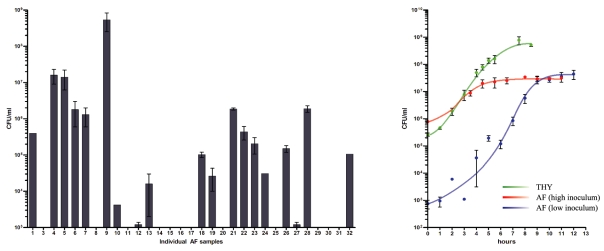
Characterization of growth of GAS in AF. **A**. Individual specimens of AF support growth of GAS at various levels after 24 h of incubation; initial inoculum ∼10^4^ CFU/ml. **B**. Growth densities of GAS (CFU/ml) in THY and AF.

To determine if AF had antimicrobial properties towards GAS, we tested samples collected from separate individuals at different stages of pregnancy. About half of the tested specimens did not support the growth of GAS (Fig 1A). Observed inhibition of GAS growth did not correlate with the gestation period (data not shown), and was instead patient specific and influenced by unknown factors. Visual inspection of AF specimens did not allow determination of the presence of meconium. Growth of GAS in AF was not restricted to the serotype M28 strain, as serotype M1 strain MGAS5005 grew at a comparable density to the M28 strain in one of the individual growth-positive specimens (data not shown). In conclusion, unlike many other bacteria, GAS can survive and grow in some AF specimens, which could have important implications for its ability to cause postpartum infections.

To further characterize the growth of GAS in AF, we pooled all AF specimens that supported growth of GAS and performed growth curves with two different starting inocula and compared it to GAS grown in laboratory medium (THY) (Fig. 1B). Interestingly, GAS grown in AF reached similar density (around 2×10^7^ CFU/ml) in stationary phase, despite a three-fold difference in starting inocula. Similar cell density was observed after 48h of incubation in AF. This suggests that the limited nutrient availability of AF could not sustain higher density growth.

### Microarray analysis: general quantitative trends in response to AF

**Figure 2:**
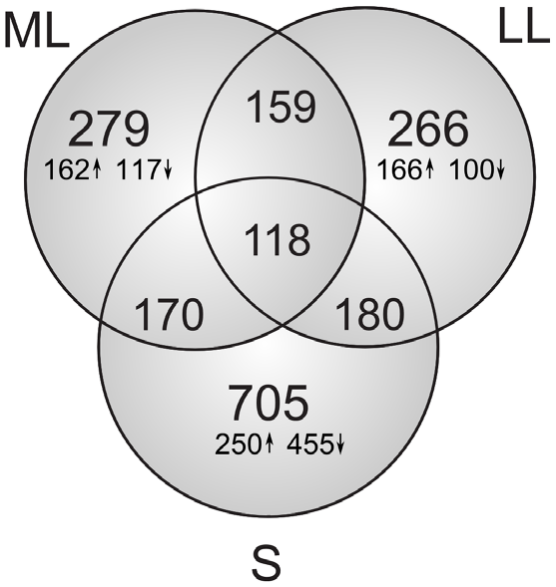
Number of genes differentially expressed in response to AF in ML, LL and S phases. Upward arrows represent the number of transcripts with increased expression in AF in ML, LL and S phases; the downward arrows represent the number of transcripts with increased expression in THY (down regulated in AF) in ML, LL and S phases. Intersections of the Venn diagram indicate number of differentially expressed genes during more than one growth phase.

**Figure 3:**
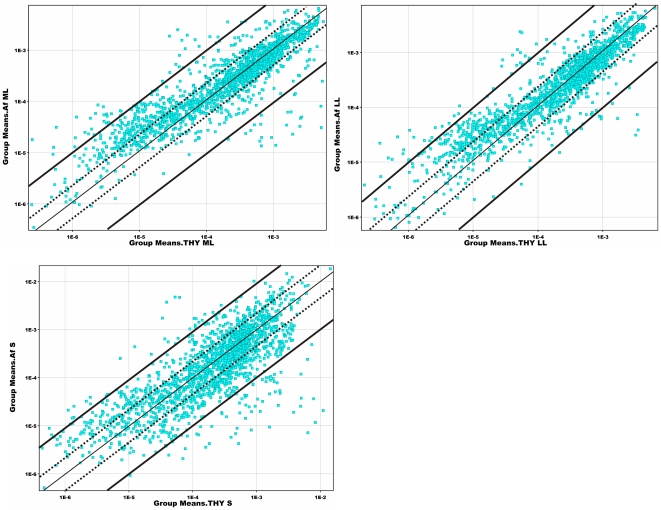
Dynamics of gene expression of GAS genes in ML, LL, and S phase in response to AF. Each dot represents a single transcript and its coordinates are the average level of expression in THY (x-axis) and AF (y-axis). The dotted lines denote 2-fold change in transcription. Transcripts below the lower dotted line are more highly expressed in THY, transcripts above the upper dotted line are more highly expressed in AF. Thick black lines denote 10-fold differences in transcript level between the studied conditions.

To characterize the transcriptional response of GAS grown in AF we utilized an ex vivo microarray approach that has been used previously to characterize the interactions of GAS with various environments such as blood and saliva [10], [11]. We detected 859 differentially expressed genes fulfilling the criteria of differentially expressed gene (∼49% of genes present on the array). Over 250 genes were differentially expressed in ML and LL phases and the number of differentially expressed genes increased and reached maximum in S phase (Figures 2 and 3). The majority of these genes in ML and LL phases were up regulated rather than down regulated. This differs from the response of GBS grown in AF [19], in which the majority of genes are down regulated when compared to laboratory conditions and the bulk of changes are observed during the transition from logarithmic to stationary phase [19]. The complete list of transcriptional changes in GAS is shown in Table S1 and comparison of gene expression between GAS and GBS in Table S2.

### Expression of virulence factors

Because GAS causes severe invasive postpartum diseases, we were interested in determining which virulence factors exhibited a change in expression in response to AF. Surprisingly, with the exception of CAMP factor, streptodornase, and one of the enterotoxins (Table 1), we did not detect up regulation of virulence factor expression. However, we did detect massive down regulation of genes encoding proteins involved in adhesion, such as multiple fibronectin, collagen and laminin binding proteins, M protein, the gene encoding R28 protein (*M28_Spy1336*), and streptolysin S. Down regulation of multiple adhesins and cell surface proteins could be a mechanism to evade the host immune response. The genes encoding two peptidases that interact with the host immune response, C5A peptidase and SpyCEP, were expressed at much lower levels in AF than in THY (Table 1). C5A peptidase has been also shown to facilitate fibronectin-independent invasion of epithelial cells [30]. Similar down regulation of adhesins and capsule was observed in GBS when grown in AF [19].

**Table 1:** Differential expression of genes encoding known GAS virulence factors upon contact with AF.

6180	SF370	Locus	ML	LL	S	Descriptions
M28_Spy0105			**−4.20**	**−12.76**	**−28.84**	Fibronectin-binding protein
M28_Spy0107		-	**−2.49**	**−9.14**	**−7.83**	Fibronectin-binding protein
M28_Spy0109	SPy0128	-	**−2.33**	**−5.79**	**−5.36**	Fibronectin-binding protein
M28_Spy0113			**−2.49**	**−2.64**	**−16.21**	Collagen adhesion protein
M28_Spy0754	SPy1054	-	**−2.41**		**−1.82**	Collagen-like surface protein
M28_Spy1675		sclA	**−167.84**	**−13.82**	**−2.83**	Collagen-like surface protein
M28_Spy1696	SPy2007	lmb		**−2.90**	**−5.03**	Laminin-binding surface protein
M28_Spy1702	SPy2018	emm	**−21.63**	**−3.84**		M protein
M28_Spy1715			**−53.54**	**−8.97**	**−3.47**	Fibronectin-binding protein
M28_Spy1716			**−75.85**	**−15.05**	**−5.01**	Fibronectin-binding protein
M28_Spy1336			**−53.13**	**−90.92**	**−133.85**	R28 protein
M28_Spy0139	SPy0167	slo	**−45.03**		**2.68**	Streptolysin O
M28_Spy0540	SPy0738	sagA			**−2.06**	Streptolysin S precursor
M28_Spy0541	SPy0739	sagB			**−12.20**	Streptolysin S biosynthesis protein
M28_Spy0542	SPy0740	sagC			**−18.65**	Streptolysin S biosynthesis protein
M28_Spy0543	SPy0741	sagD			**−15.04**	Streptolysin S biosynthesis protein
M28_Spy0544	SPy0742	sagE			**−15.65**	Streptolysin S biosynthesis protein
M28_Spy0545	SPy0743	sagF			**−15.06**	Streptolysin S biosynthesis protein
M28_Spy0546	SPy0744	sagG			**−18.88**	Streptolysin S export ATP-binding protein
M28_Spy0547	SPy0745	sagH			**−16.89**	Streptolysin S export protein
M28_Spy0548	SPy0746	sagI			**−14.21**	Streptolysin S export protein
M28_Spy0180	SPy0212	speG			**−2.59**	Enterotoxin
M28_Spy0969			**2.53**			Enterotoxin
M28_Spy0953	SPy1273	cfa	**15.18**	**2.52**		cAMP factor
M28_Spy0968	SPy1436	spd3	**2.26**			Streptodornase (EC 3.1.21.1)
M28_Spy0329	SPy0416	Spy CEP	**−16.21**		**−2.50**	Endopeptidase lactocepin
M28_Spy1700	SPy2010	scpA	**−47.34**	**−2.64**		C5A peptidase precursor (EC 3.4.21.-)

Values represent fold change in expression in AF at ML, LL, and S growth phases compared to expression in THY medium.

R28 is an adhesin encoded by the RD2 element present in MGAS6180 and several other GAS strains [5]. It is a known virulence factor that increases attachment of GAS to cervical cells [31]. Its down regulation might be connected to down regulation of *M28_Spy1337*, which encodes a putative regulator; however, this has yet to be tested experimentally. In addition to R28, we observed an increase in transcription levels for multiple genes encoded by RD2 (Table 2). The RD2 element has been suggested to be involved in pathogenesis and adaptation to specific environments [5]. Interestingly, a large number of RD2 genes differed in their expression among biological replicates; therefore the calculated P value in many cases was greater than 0.05.

**Table 2:** Differential expression of RD2 genes in response to AF.

Gene	ML	P	LL		S		Putative function	note
M28_Spy1304	**10.19**		**6.76**		**16.10**	+	Hypothetical Protein	
M28_Spy1305	**2.39**	+	**2.43**	+	**1.75**	+	Hypothetical membrane protein	
M28_Spy1306	**10.06**	+	**7.60**	+	**2.47**		Cell surface protein	Virulence
M28_Spy1307	**5.13**		**3.98**	+	**1.08**		Hypothetical exported protein	Virulence
M28_Spy1308	**5.52**		**2.65**		**3.81**	+	Hypothetical exported protein	Virulence
M28_Spy1309	**1.54**	+	**1.71**	+	**1.70**	+	Hypothetical protein	
M28_Spy1310	**3.41**		**3.13**	+	**1.78**		Membrane protein	
M28_Spy1311	Not detected							
M28_Spy1312	Not detected							
M28_Spy1313	**8.70**		**6.23**		**2.28**		Hypothetical membrane protein	
M28_Spy1314	**5.17**		**4.37**	+	**3.00**		hypothetical protein	
M28_Spy1315	**5.69**		**7.31**	+	**3.42**		Hypothetical protein	
M28_Spy1316	**9.67**		**8.45**		**6.03**		Hypothetical protein	
M28_Spy1317	**35.90**		**12.95**		**3.67**		Hypothetical protein	
M28_Spy1318	**3.81**		**2.69**		**6.31**	+	Hypothetical protein	
M28_Spy1319	Not detected							
M28_Spy1320	**11.20**		**36.55**	+	**10.89**	+	Hypothetical cytosolic protein	
M28_Spy1321	Not detected							
M28_Spy1322	**6.26**		**4.41**		**2.37**		FtsK SpoIIIE family	
M28_Spy1323	**9.05**	+	**6.88**	+	**1.61**		Hypothetical cytosolic protein	
M28_Spy1324	**5.80**		**7.41**	+	**2.68**		Hypothetical cytosolic protein	
M28_Spy1325	**3.99**	+	**2.28**		**1.72**		Cell surface protein	Virulence
M28_Spy1326	**2.18**	+	**1.08**		**−1.29**	+	M like protein	Virulence
M28_Spy1327	**18.65**		**6.43**		**4.84**		Hypothetical cytosolic protein	
M28_Spy1328	Not detected							
M28_Spy1329	**12.91**		**5.16**		**2.98**		Transcriptional Cro CI regulator	
M28_Spy1330	**1.80**	+	**1.75**	+	**2.23**	+	Transcriptional Cro CI regulator	
M28_Spy1331	**2.22**	+	**2.51**	+	**4.28**	+	Hypothetical cytosolic protein	
M28_Spy1332	**2.42**	+	**2.50**	+	**8.66**	+	Hypothetical exported protein	Virulence
M28_Spy1333	**1.89**	+	**1.69**	+	**5.46**	+	Hypothetical cytosolic protein	
M28_Spy1334	**1.74**	+	**2.35**	+	**6.34**	+	DNA-damage-inducible protein J	
M28_Spy1335	**1.20**		**1.36**		**3.20**		Transposase	
M28_Spy1336	**−53.13**	+	**−90.92**	+	**−133.85**	+	R28 Cell surface protein	Virulence
M28_Spy1337	**−5.18**	+	**−8.50**	+	**−7.93**	+	Transcriptional regulator	

Values represent fold change in expression in AF at ML, LL, and S growth phases compared to expression in THY medium. +: P<0.05.

### Stress response of GAS and GBS to AF

In contrast to GBS, which does not increase transcription of genes encoding proteins involved in the stress response [19], GAS activates multiple genes involved in this process. We observed increased transcription of the genes encoding proteolytic complex composed of ATPase and catalytic subunits ( *clpP*, *clpE*, *clpX*, and *clpL*) known to be stress effectors in streptococci [32], [33], the GroEL and GroES chaperonins, members of the Gls24 family of stress proteins (stress and starvation inducible genes in *Enterococcus faecalis*, [34]), and a putative GTP pyrophosphokinase (Table 3). Expression of the gene *M28_Spy1562* that encodes a putative stress protein, was dramatically decreased in AF; however, its function in GAS is unknown. We also observed lowered expression of the *relA* gene which is major regulator of stringent control in GAS [35].

**Table 3:** Differential expression of stress response genes in response to AF.

6180	SF370	Locus	ML	LL	S	Descriptions
M28_Spy0317	SPy0395	clpP			**3.01**	proteolytic subunit clpP
M28_Spy1179	SPy1509	clpE	**2.01**	**2.64**	**2.04**	ATP-binding subunit clpE
M28_Spy0659	SPy0873	-			**2.05**	GTP pyrophosphokinase
M28_Spy0671		clpX			**−6.23**	ATP-binding subunit clpX
M28_Spy0674	SPy0888	clpL	**3.29**	**6.32**		ATP-binding subunit clpL
M28_Spy0943	SPy1260	-	**2.66**			General stress protein, Gls24 family
M28_Spy0945	SPy1262	-	**2.59**			General stress protein, Gls24 family
M28_Spy1286	SPy1557	msrA		**3.73**	**2.41**	Peptide methionine sulfoxide reductase msrA
M28_Spy0755	SPy1055	msrB	**5.36**	**4.95**	**8.23**	Peptide methionine sulfoxide reductase msrB
M28_Spy1503	SPy1780	-			**2.71**	Universal stress protein family
M28_Spy1562		-		**−102.42**	**−17.90**	Universal stress protein family
M28_Spy0659	SPy0873	-			**2.05**	GTP pyrophosphokinase
M28_Spy1674	SPy1981	relA	**−2.11**		**−2.15**	GTP pyrophosphokinase
M28_Spy1747	SPy2070	groEL			**2.34**	60 kDa chaperonin GROEL
M28_Spy1748	SPy2072	groES			**2.36**	10 kDa chaperonin GROES
M28_Spy1751	SPy2077	csp		**2.24**	**8.50**	Cold shock protein

Values represent fold change in expression in AF at ML, LL, and S growth phases compared to expression in THY medium.

### Regulatory events during growth in AF

We observed a large number of significant changes in transcription of regulatory genes (Table S1). Because the exact functions of many of these are unknown, it is impossible to predict the significance of these changes. However, we did detect changes in expression of known GAS regulators (Table 4) such as RofA/RALP that are involved in the regulation of virulence factor expression, namely adhesins [36]. Down regulation of *rofA* in response to AF might be partially responsible for the observed decreases in the transcription of the genes encoding fibronectin binding proteins. The decreased gene expression of the regulator *ahrC.2* may be responsible for the observed changes in expression of genes involved in arginine metabolism (see below). Two other differentially expressed regulators were recently shown to be involved in GAS pathogenesis. The first, MtsR, is a regulator of ion transport and is involved in the development of necrotizing fasciitis [37], [38] The second, CcpA, links GAS virulence and carbohydrate metabolism [18]. In addition to individual regulators, we detected differential expression of seven (of 13) two component systems (TCSs) encoded by GAS. In the absence of regulation by alternative sigma factors in GAS, it is believed that concerted activity of regulons and TCSs is responsible for reaction of GAS to the environment. FasBCA and ihk/irr were shown to be involved in regulation of GAS pathogenesis. FasBCA controls expression of streptokinase, streptolysin S, and fibronectin binding proteins [39], [40]. The Ihk/Irr system is involved in GAS survival upon contact with human PMNs [12]. Circuits regulated by two other two component systems, M28_Spy0761/2 (SPy1061/2 in SF370 strain) and M28_Spy0807/8 (SPy1106/7 in SF370 strain), were studied by microarray analysis [17] and were shown to be involved in regulation of genes involved in carbohydrate and malate utilization, respectively. M28_Spy0919/20, M28_Spy1346/7 and M28_Spy1373/4 (SPy1236/7, SPy1587/8, SPy1621/2 in SF370 strain, respectively) have not been characterized thus far.

**Table 4:** Differential expression of selected regulatory systems in response to AF.

6180	SF370	Locus	ML	LL	S	Descriptions
M28_Spy0104	SPy0124	rofA	**−2.09**	**−3.56**	**−2.69**	Transcriptional regulator RofA
M28_Spy0122	SPy0146	sloR	**−11.53**	**−13.76**		Transcriptional regulator pfoR
M28_Spy0198	SPy0242	fasB			**−3.31**	Sensory transduction protein kinase FasB
M28_Spy0199	SPy0244	fasC	**−2.08**	**−2.09**	**−4.77**	Sensory transduction protein kinase FasC
M28_Spy0200	SPy0245	fasA			**−3.61**	Response regulator FasA
M28_Spy0356	SPy0450	mtsR			**3.96**	Iron-dependent repressor
M28_Spy0412	SPy0514	ccpA			**−3.65**	Catabolite control protein A
M28_Spy0465	SPy0584	ptsK			**2.66**	HPR(SER) kinase
M28_Spy0761	SPy1061	-			**−4.83**	Two-component sensor kinase yesM
M28_Spy0762	SPy1062	-			**−3.19**	Two-component response regulator yesN
M28_Spy0807	SPy1106	dpiA	**2.09**			Transcriptional regulatory protein dpiA
M28_Spy0808	SPy1107	dpiB	**2.16**			Sensor kinase dpiB
M28_Spy0919	SPy1236	ciaH			**−3.35**	Sensor protein ciaH
M28_Spy0920	SPy1237	ciaR			**−2.58**	Transcriptional regulatory protein ciaR
M28_Spy1215	SPy1549	ahrC.2			**−16.22**	Arginine repressor, argR
M28_Spy1346	SPy1587	-			**−4.45**	Two-component response regulator yesN
M28_Spy1347	SPy1588	-			**−3.03**	Two-component sensor kinase yesM
M28_Spy1373	SPy1621	yvqC			**2.05**	Two-component response regulator yvqC
M28_Spy1374	SPy1622	yvqE		**2.28**	**3.93**	Two-component sensor protein yvqE
M28_Spy1708	SPy2026	ihk			**−1.96**	Two component system histidine kinase
M28_Spy1709	SPy2027	irr			**−1.58**	Two-component response regulator

Values represent fold change in expression in AF at ML, LL, and S growth phases compared to expression in THY medium.

### Metabolic adaptation to AF environment

A recently performed transcriptional analysis of GBS grown in AF revealed dramatic changes in expression of genes that control metabolism of carbohydrates, amino acids, and nucleotides [19], which presumably reflects an adaptive response to limited nutrient availability. We observed similar trends in the metabolic response of GAS to AF, with the majority of differences occurring in expression of genes involved in carbohydrate utilization (Table S2). In contrast to GBS that up regulates genes involved in carbohydrate utilization, GAS down regulates genes belonging to this category. Clear examples are the M28_Spy1036-1048 (*mal* genes), M28_Spy1349-1354, M28_Spy1438-1445 (*lac.1* genes), and M28_Spy1622-1629 (*lac.2* genes) loci (Table 5). The Mal locus has been shown to be involved in GAS persistence in the oropharynx [41], and *lac* loci are not only involved in fermentation and energy production via the tagatose pathway, but also link metabolism and the regulation of virulence [42].

**Table 5:** Differential expression of selected genes responsible for transport and metabolism of carbohydrates.

6180	SF370	Locus	ML	LL	S	Descriptions
M28_Spy0757	SPy1057	-	**−3.86**	**−11.47**	**−18.53**	PTS system, mannose fructose family
M28_Spy0759	SPy1059	ptsC	**−4.04**	**−7.65**	**−13.42**	PTS system, mannose fructose family
M28_Spy0958	SPy1280	glmS	**−2.80**	**−2.01**		Glucosamine–fructose-6-phosphate aminotransferase
M28_Spy1036	SPy1291	glgP			**−2.16**	Maltodextrin phosphorylase
M28_Spy1037	SPy1292	malM			**−14.96**	4-alpha-glucanotransferase
M28_Spy1039	SPy1294	malE			**−4.94**	Maltose maltodextrin-binding protein
M28_Spy1041	SPy1296	malG	**2.19**			Maltose transport system permease protein
M28_Spy1044	SPy1299	malD			**−34.73**	Maltodextrin transport system permease protein
M28_Spy1045	SPy1301	malC			**−38.81**	Maltodextrin transport system permease protein
M28_Spy1046	SPy1302	amyA			**−82.63**	Cyclodextrin glucanotransferase
M28_Spy1047	SPy1304	amyB	**2.06**		**−24.86**	Neopullulanase
M28_Spy1048	SPy1306	malX			**−37.14**	Maltose maltodextrin-binding protein
M28_Spy1349	SPy1592	-			**−42.74**	Sugar-binding protein
M28_Spy1350	SPy1593	-			**−13.77**	Sugar transport system permease protein
M28_Spy1351	SPy1595	-			**−7.07**	Sugar transport system permease protein
M28_Spy1354	SPy1599	-			**−2.17**	Beta-glucosidase
M28_Spy1438	SPy1704	lacD.1			**−49.45**	Tagatose-bisphosphate aldolase
M28_Spy1440	SPy1707	lacB.1			**−170.54**	Galactose-6-phosphate isomerase lacB subunit
M28_Spy1441	SPy1708	lacA.1			**−100.49**	Galactose-6-phosphate isomerase lacA subunit
M28_Spy1442	SPy1709	-	**2.40**			PTS system, galactose-specific IIC component
M28_Spy1443	SPy1710	-			**−26.62**	PTS system, galactose-specific IIB component
M28_Spy1444	SPy1711	-			**−57.34**	PTS system, galactose-specific IIA component
M28_Spy1445	SPy1712	lacR.1			**−2.47**	Lactose phosphotransferase system repressor
M28_Spy1622	SPy1916	lacG			**−67.36**	6-phospho-beta-galactosidase
M28_Spy1624	SPy1918	lacF			**−8.85**	PTS system, lactose-specific IIA component
M28_Spy1625	SPy1919	lacD.2			**−122.01**	Tagatose-bisphosphate aldolase
M28_Spy1626	SPy1921	lacC.2			**−55.33**	Tagatose-6-phosphate kinase
M28_Spy1627	SPy1922	lacB.2			**−21.89**	Galactose-6-phosphate isomerase lacB subunit
M28_Spy1628	SPy1923	lacA.2			**−69.34**	Galactose-6-phosphate isomerase lacA subunit
M28_Spy1629	SPy1924	lacR.2			**−3.72**	Lactose phosphotransferase system repressor
M28_Spy1668	SPy1972	pulA			**−5.30**	Pullulanase
M28_Spy1767	SPy2096	dexS		**−6.18**	**−39.01**	Trehalose-6-phosphate hydrolase
M28_Spy1768	SPy2097	-		**−8.81**	**−43.06**	PTS system, trehalose-specific IIBC component

Values represent fold change in expression in AF at ML, LL, and S growth phases compared to expression in THY medium.

Another category of metabolic genes affected by growth in AF were genes involved in protein and amino acid utilization. We observed differential expression of multiple proteases, and di- and oligopeptide transport systems (Table 6). Similarly to GBS, we observed up regulation of genes involved in uptake and utilization of branched chain amino acids (BCAA, valine, leucine, and isoleucine). GBS is auxotrophic in respect to BCAA and mobilizes all transport systems to maximize utilization from AF [19]; GAS could possibly employ the same mechanism to maximize nutrient utilization.

**Table 6:** Differential expression of genes involved in the transport and metabolism of amino acids, peptides, and amines.

6180	SF370	Locus	ML	LL	S	Descriptions
Proteases						
M28_Spy0463	SPy0581	-			**−2.95**	Metallopeptidase, SprT family
M28_Spy0470	SPy0590	-		**−2.23**	**−2.82**	Peptidase family U32
M28_Spy0471	SPy0591	-			**−2.10**	Peptidase family U32
M28_Spy0572	SPy0775	-			**2.31**	Neutral zinc metallopeptidase family
M28_Spy1136	SPy1402	-			**34.77**	Peptidase family S11
M28_Spy1397	SPy1651	pepC	**−1.98**			Aminopeptidase C
M28_Spy1565	SPy1858	pepXP		**2.31**	**4.45**	Xaa-Pro dipeptidyl-peptidase
M28_Spy1744	SPy2066	-		**−4.28**	**−22.44**	Dipeptidase A (EC 3.4.13.-)
M28_Spy1766	SPy2095	pepO	**−2.78**			Oligoendopeptidase O (EC 3.4.24.-)
Arginine metabolism						
M28_Spy0575	SPy0778	-			**−2.35**	Arginine-binding protein
M28_Spy1176	SPy1506	artP	**2.95**	**2.12**	**2.07**	Arginine transport ATP-binding protein
M28_Spy1177	SPy1507	artQ	**2.50**	**2.08**		Arginine transport system permease protein
M28_Spy1208	SPy1541	arcC		**−8.01**	**−877.27**	Carbamate kinase
M28_Spy1209	SPy1542	-			**−199.49**	Xaa-His dipeptidase
M28_Spy1210	SPy1543	-		**−2.55**	**−191.92**	Arginine ornithine antiporter
M28_Spy1211	SPy1544	arcB		**−4.96**	**−212.09**	Ornithine carbamoyltransferase
M28_Spy1213	SPy1547	arcA		**−8.87**	**−712.24**	Arginine deiminase
Branched chain amino acids						
M28_Spy0266	SPy0323	braB	**5.13**	**5.26**	**2.21**	Branched-chain amino acid carrier protein
M28_Spy0693	SPy0911	bcaT		**2.13**	**3.48**	Branched-chain amino acid aminotransferase
Di- and oligopeptide transport						
M28_Spy1689	SPy2000	dppA	**−4.23**	**−2.68**		Dipeptide-binding protein
M28_Spy1690	SPy2001	dppB	**−3.39**	**−2.70**		Dipeptide transport system permease protein
M28_Spy1691	SPy2002	dppC	**−4.57**	**−2.82**	**−2.61**	Dipeptide transport system permease
M28_Spy1692	SPy2003	dppD	**−4.19**	**−2.58**	**−2.46**	Dipeptide transport ATP-binding protein
M28_Spy1693	SPy2004	dppE	**−4.21**	**−2.76**		Dipeptide transport ATP-binding protein
M28_Spy0245	SPy0294	oppB		**2.55**		Oligopeptide transport system permease
M28_Spy0246	SPy0295	oppC		**2.01**		Oligopeptide transport system permease
M28_Spy0247	SPy0296	oppD		**1.75**		Oligopeptide transport ATP-binding
M28_Spy0248	SPy0297	oppF		**1.89**		Oligopeptide transport ATP-binding

Values represent fold change in expression in AF at ML, LL, and S growth phases compared to expression in THY medium.

The most dramatic changes in gene expression were observed in genes encoding enzymes of the arginine deiminase pathway, which were down regulated almost 900-fold upon contact with AF (Table 6). The arginine deiminase pathway allows utilization of arginine as a carbon and energy source [43] and plays a role in the modification of the environmental acidity [44], [45]. Arginine deiminase from the GAS Manfredo strain is also a potent inhibitor of the proliferation of peripheral blood mononuclear cells [46]. However, arginine deiminase is not only an intracellular enzyme involved in the aforementioned processes. It can be also found on the GAS cell surface [47] and antibodies against this protein have been recently reported to protect mice against GAS infection (Walker MJ, Henningham A, Cork A, Cole JN, Ramachandran V et al. (2008) Group A Anchorless Surface proteins. XVII Lancefield International Symposium on Streptococci and Streptococcal Diseases O9.2; Henningham A, Batzloff M, Cole JN, Gillen C, Hartas J et al. (2008) Protection Against lethal *Streptococcus pyogenes* challenge following vaccination with anchorless cell wall-associated proteins. XVII Lancefield International Symposium on Streptococci and Streptococcal Diseases P54). It is possible that the lack of arginine deiminase on the surface is linked to decreased recognition by the host immune response, which would be consistent with the observed down regulation of adhesins and other major surface proteins. Interestingly, fetal urine (which is a major component of AF) flow and composition is modulated by arginine levels [22], therefore bacterial arginine metabolism could be linked to AF dynamics.

**Figure 4:**
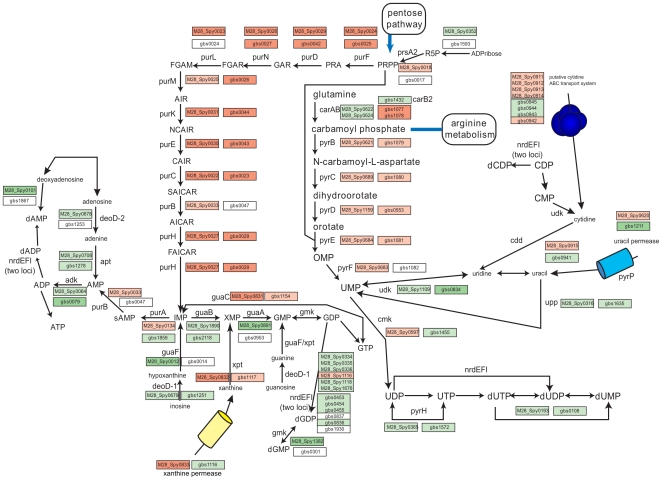
Changes in transcription of genes encoding predicted enzymes in the purine and pyrimidine biosynthetic pathway in GAS and GBS in response to AF. Both GAS and GBS exhibit similar changes in the expression of genes encoding enzymes involved in nucleotide biosynthesis. Changes in transcription of GBS genes from [19], changes in transcription of GAS genes, this work. Light green: genes down regulated 2 to 5 fold upon contact with AF; dark green: above 5 fold. Light orange: genes up regulated 2 to 5 fold upon contact with AF; dark orange: above 5 fold. R5P, ribose 5-phosphate; PRPP, phosphoribosylpyrophosphate; PRA, 5-phospho-b-D-ribosylamine; GAR, 5′-phosphoribosylglycinamide; FGAR, 5′-phosphoribosyl-N-formylglycinamide; FGAM, 5′-phosphoribosyl-N-formylglycinamidine; AIR, 5′-phosphoribosyl-5-aminoimidazole; NCAIR, 5′-phosphoribosyl-5-carboxyaminoimidazole; CAIR, 5′-phosphoribosyl-5-aminoimidazole-4-carboxylate; SAICAR, 5′-phosphoribosyl-4-(N-succino-carboxamide)-5-aminoimidazole; AICAR, 5′-phosphoribosyl-4-carboximide-5-aminoimidazole ribonucleotide; FAICAR, 5′-phosphoribosyl-4-carboximide-5-formaminoimidazole; IMP, inosine 5′-monophosphate; XMP, xanthosine monophosphate; GMP, guanosine monophosphate; GTP, guanosine triphosphate; sAMP, adenylosuccinate; AMP, adenosine monophosphate; ATP, adenosine triphosphate.

Arginine metabolism is linked via carbamate kinase to another pathway – nucleotide synthesis (Figure 4). We observed dramatic up regulation of genes involved in purine and pyrimidine nucleotide biosynthesis pathways and down regulation of genes encoding enzymes involved in salvage pathways (Table 7). The extent of changes in GAS correlates well with changes detected in GBS [19], and almost all of the enzymes in the nucleotide metabolic pathways undergo the same directional changes (Figure 4), what suggests direct influence of the AF environment.

**Table 7:** Differential expression of selected genes involved in nucleotide metabolism upon contact with AF.

6180	SF370	Locus	ML	LL	S	Descriptions
M28_Spy0022	SPy0024	-	**36.60**	**10.73**	**35.84**	Phosphoribosylaminoimidazole-succinocarboxamide synthase
M28_Spy0024	SPy0026	purF	**24.03**	**10.89**	**13.45**	Amidophosphoribosyltransferase
M28_Spy0026	SPy0028	purN	**37.86**	**18.63**	**17.69**	Phosphoribosylglycinamide formyltransferase
M28_Spy0027		purH	**17.83**	**3.68**	**3.85**	Phosphoribosylaminoimidazolecarboxamide formyltransferase
M28_Spy0029	SPy0032	purD	**15.56**	**9.89**	**11.31**	Phosphoribosylamine-glycine ligase
M28_Spy0030	SPy0033	purE	**16.52**	**7.50**	**11.42**	Phosphoribosylaminoimidazole carboxylase carboxyltransferase
M28_Spy0031	SPy0034	purK		**6.05**	**11.19**	Phosphoribosylaminoimidazole carboxylase NCAIR mutase
M28_Spy0064	SPy0074	adk			**−8.25**	Adenylate kinase
M28_Spy0134	SPy0160	purA			**3.17**	Adenylosuccinate synthetase
M28_Spy0193	SPy0235	-			**−2.67**	Deoxyuridine 5 -triphosphate nucleotidohydrolase
M28_Spy0316	SPy0392	upp			**−2.43**	Uracil phosphoribosyltransferase
M28_Spy0334	SPy0425	nrdF.1	**−5.27**			Ribonucleoside-diphosphate reductase beta chain
M28_Spy0335	SPy0426	nrdI	**−4.58**			NrdI protein
M28_Spy0336	SPy0427	nrdE.1	**−2.37**			Ribonucleoside-diphosphate reductase alpha chain
M28_Spy0597	SPy0803	cmk			**2.59**	Cytidylate kinase
M28_Spy0620	SPy0831	pyrP	**6.66**			Uracil permease
M28_Spy0622	SPy0833	carA	**4.79**		**−2.17**	Carbamoyl-phosphate synthase small chain
M28_Spy0624	SPy0835	carB	**5.93**		**−2.87**	Carbamoyl-phosphate synthase large chain
M28_Spy0658	SPy0872	-	**68.19**	**15.18**	**11.50**	5 -nucleotidase
M28_Spy0668	SPy0882	thyA			**−3.66**	Thymidylate synthase
M28_Spy0679	SPy0894	deoD2			**−2.85**	Purine nucleoside phosphorylase
M28_Spy0683	SPy0900	pyrF	**3.45**			Orotidine 5 -phosphate decarboxylase
M28_Spy0684	SPy0901	pyrE	**4.03**			Orotate phosphoribosyltransferase
M28_Spy0689	SPy0907	pyrC	**2.82**	**2.39**	**2.51**	Dihydroorotase
M28_Spy0708	SPy0927	apt			**−2.13**	Adenine phosphoribosyltransferase
M28_Spy0821	SPy1123	-			**2.04**	Ribose-phosphate pyrophosphokinase
M28_Spy0831	SPy1135	guaC	**68.20**	**23.60**	**95.96**	GMP reductase
M28_Spy0832	SPy1136	xpt		**40.85**	**90.15**	Xanthine phosphoribosyltransferase
M28_Spy0833	SPy1137	-	**9.77**	**45.75**	**76.46**	Xanthine permease
M28_Spy0897	SPy1211	rnhB		**2.92**	**8.45**	Anaerobic ribonucleoside-triphosphate reductase
M28_Spy0899	SPy1213	fhs.1	**5.53**	**4.21**		Formate–tetrahydrofolate ligase
M28_Spy0914	SPy1228	-			**2.11**	Nucleoside-binding protein
M28_Spy1109	SPy1368	udk			**−2.04**	Uridine kinase
M28_Spy1118	SPy1378	nrdF.2			**2.32**	Ribonucleoside-diphosphate reductase beta chain
M28_Spy1159	SPy1432	pyrD		**2.15**		Dihydroorotate dehydrogenase
M28_Spy1466	SPy1736	-	**5.01**	**3.02**	**5.14**	Guanine-hypoxanthine permease
M28_Spy1580	SPy1869	udp			**−2.83**	Uridine phosphorylase
M28_Spy1599	SPy1894	pyrG			**−2.64**	CTP synthase
M28_Spy1758	SPy2085	fhs.2	**3.32**	**2.32**		Formate–tetrahydrofolate ligase
M28_Spy1773	SPy2105	nrdG	**−2.00**	**−2.45**		Anaerobic ribonucleoside-triphosphate reductase activating protein
M28_Spy1777	SPy2110	nrdD	**−2.22**	**−3.50**		Anaerobic ribonucleoside-triphosphate reductase
M28_Spy1890	SPy2206	guaB			**−2.28**	Inosine-5 -monophosphate dehydrogenase

Values represent fold change in expression in AF at ML, LL, and S growth phases compared to the expression in THY medium.

**Figure 5:**
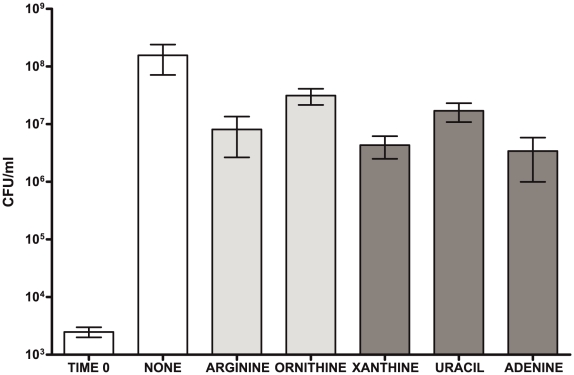
Addition of arginine, ornithine, purines and pirymidines does not increase growth of GAS in AF. Cell densities of GAS cultures grown in pooled AF for 24 hours with addition of amino acids (arginine, ornithine) and nucleotides (xanthine, uracil, and adenine) at the concentration that supports growth of GAS in minimal CDM [49].

Samant et al. [48] recently showed that multiple enzymes involved in nucleotide metabolism are essential for growth of *Escherichia coli* in human serum, and a bacterial growth defect can be rescued by the addition of nucleotides to the serum. To test if a lack of nucleotides or arginine/ornithine in the AF is a growth limiting factor, we tested growth of GAS in AF with the addition of selected amino acids and nucleotides (arginine, ornithine, adenine, xanthine, and uracyl at concentrations corresponding to minimal chemically defined media (CDM) concentrations for GAS [49]). The observed culture densities after overnight growth did not increase compared to the AF without any supplements (Figure 5). Therefore, the lack of arginine and nucleotides in AF is not a limiting factor for growth of GAS and up regulation of the nucleotide synthesis pathway has a distinct function from providing material for DNA synthesis. Multiple reports have suggested a connection between nucleotide metabolism and bacterial virulence [50]. Mereghetti et al. recently reported changes in nucleotide metabolism in GBS during growth in blood and as an effect of a temperature switch [51], [52].

### Summary

Group A *Streptococcus*, a causative agent of postpartum invasive infections, is able to survive and multiply in AF. It is able to multiply and survive over 48h in the AF environment. The response of GAS is in many aspects similar to the response exhibited by GBS; however, on the contrary to GBS, GAS exhibits stronger stress response to the AF environment. GAS adapts to growth in AF by differential regulation of genes involved in arginine and nucleotide metabolism. In addition, GAS down regulates multiple cell surface proteins, presumably to escape host immune recognition.

## Supporting Information

Table S1 Differential expression of all GAS genes in response to AF. Changes in transcription of GAS genes upon contact with amniotic fluid. All changes detected in transcription of GAS in response to amniotic fluid. Values represent fold change in expression in amniotic fluid compared to expression in THY; ML, mid-logarithmic growth phase; LL, late-logarithmic growth phase; S, stationary growth phase; cut-off two fold change with P value less than 0.05. Positive values represent genes up-regulated in AF (orange), negative values represent down-regulated (green, better expressed in THY) genes. Rows marked grey denote transcripts not detected during the experiment.
(0.62 MB XLS)

Table S2 Comparison of transcriptional changes between GAS and GBS grown in AF. Differential expression of GAS and GBS genes in response to AF - Changes in transcription of GAS and GBS genes upon contact with amniotic fluid. Values represent fold change in expression in amniotic fluid compared to expression in THY; ML, mid-logarithmic growth phase; LL, late-logarithmic growth phase; S, stationary growth phase; cut-off two fold change with P value less than 0.05. Positive values represent genes up-regulated in AF (orange), negative values represent down-regulated (green, better expressed in THY) genes. GBS data after reference [19].
(0.34 MB XLS)

## References

[1] Cunningham MW (2000). Pathogenesis of group A streptococcal infections.. Clin Microbiol Rev.

[2] Carapetis JR, Steer AC, Mulholland EK, Weber M (2005). The global burden of group A streptococcal diseases.. Lancet Infect Dis.

[3] Pasteur L (1879). Septicemie puerperale.. Bulletin de l'Academie de Medecine.

[4] Chuang I, Van BC, Beall B, Schuchat A (2002). Population-based surveillance for postpartum invasive group A *Streptococcus* infections, 1995–2000.. Clin Infect Dis.

[5] Green NM, Zhang S, Porcella SF, Nagiec MJ, Barbian KD (2005). Genome sequence of a serotype M28 strain of group A *Streptococcus*: potential new insights into puerperal sepsis and bacterial disease specificity.. J Infect Dis.

[6] Green NM, Beres SB, Graviss EA, Allison JE, McGeer AJ (2005). Genetic diversity among type emm28 group A *Streptococcus* strains causing invasive infections and pharyngitis.. J Clin Microbiol.

[7] Tettelin H, Masignani V, Cieslewicz MJ, Donati C, Medini D (2005). Genome analysis of multiple pathogenic isolates of *Streptococcus agalactiae*: implications for the microbial “pan-genome”.. Proc Natl Acad Sci U S A.

[8] Mylvaganam H, Bruun T, Vindenes HA, Langeland N, Skrede S (2009). Molecular epidemiological investigation of an outbreak of invasive beta-haemolytic streptococcal infection in western Norway.. Clin Microbiol Infect.

[9] Pinho MD, Melo-Cristino J, Ramirez M (2006). Clonal relationships between invasive and noninvasive Lancefield group C and G streptococci and emm-specific differences in invasiveness.. J Clin Microbiol.

[10] Graham MR, Virtaneva K, Porcella SF, Barry WT, Gowen BB (2005). Group A *Streptococcus* transcriptome dynamics during growth in human blood reveals bacterial adaptive and survival strategies.. Am J Pathol.

[11] Shelburne SA, Sumby P, Sitkiewicz I, Granville C, DeLeo FR (2005). Central role of a bacterial two-component gene regulatory system of previously unknown function in pathogen persistence in human saliva.. Proc Natl Acad Sci U S A.

[12] Voyich JM, Sturdevant DE, Braughton KR, Kobayashi SD, Lei B (2003). Genome-wide protective response used by group A *Streptococcus* to evade destruction by human polymorphonuclear leukocytes.. Proc Natl Acad Sci U S A.

[13] Goldmann O, von Kockritz-Blickwede M, Holtje C, Chhatwal GS, Geffers R (2007). Transcriptome analysis of murine macrophages in response to infection with *Streptococcus pyogenes* reveals an unusual activation program.. Infect Immun.

[14] Eidelman AI, Nevet A, Rudensky B, Rabinowitz R, Hammerman C (2002). The effect of meconium staining of amniotic fluid on the growth of *Escherichia coli* and group B *Streptococcus*.. J Perinatol.

[15] Pommerenke WT, Taylor PW (1953). Antibacterial properties of vaginal and cervical secretions and amniotic fluid.. Ann Ostet Ginecol.

[16] Sumby P, Porcella SF, Madrigal AG, Barbian KD, Virtaneva K (2005). Evolutionary origin and emergence of a highly successful clone of serotype M1 group A *Streptococcus* involved multiple horizontal gene transfer events.. J Infect Dis.

[17] Sitkiewicz I, Musser JM (2006). Expression microarray and mouse virulence analysis of four conserved two-component gene regulatory systems in group A *Streptococcus*.. Infect Immun.

[18] Shelburne SA, Keith D, Horstmann N, Sumby P, Davenport MT (2008). A direct link between carbohydrate utilization and virulence in the major human pathogen group A *Streptococcus*.. Proc Natl Acad Sci U S A.

[19] Sitkiewicz I, Green NM, Guo N, Bongiovanni AM, Witkin SS (2009). Transcriptome adaptation of group B *Streptococcus* to growth in human amniotic fluid.. PLoS One.

[20] Sozanskii A (1961). The biochemical composition of amniotic fluid and of maternal and fetal blood at various periods of pregnancy.. Biull Eksp Biol Med.

[21] Nilsson S, Ramstrom M, Palmblad M, Axelsson O, Bergquist J (2004). Explorative study of the protein composition of amniotic fluid by liquid chromatography electrospray ionization Fourier transform ion cyclotron resonance mass spectrometry.. J Proteome Res.

[22] Modena AB, Fieni S (2004). Amniotic fluid dynamics.. Acta Biomed.

[23] Beck A, Bergner-Rabinowitz S, Ofek I (1969). Effect of pH on in vitro phagocytosis of Streptococcus pyogenes.. J Bacteriol.

[24] Galask RP, Snyder IS (1970). Antimicrobial factors in amniotic fluid.. Am J Obstet Gynecol.

[25] Larsen B, Snyder IS, Galask RP (1974). Bacterial growth inhibition by amniotic fluid. I. In vitro evidence for bacterial growth-inhibiting activity.. Am J Obstet Gynecol.

[26] Larsen B, Snyder IS, Galask RP (1974). Bacterial growth inhibition by amniotic fluid. 2. Reversal of amniotic fluid bacterial growth inhibition by addition of a chemically defined medium.. Am J Obstet Gynecol.

[27] Evans HE, Levy E, Glass L (1977). Effect of amniotic fluid on bacterial growth.. Obstet Gynecol.

[28] Ahn YJ, Park SK, Oh JW, Sun HY, Shin SH (2004). Bacterial growth in amniotic fluid is dependent on the iron-availability and the activity of bacterial iron-uptake system.. J Korean Med Sci.

[29] Tran SH, Caughey AB, Musci TJ (2003). Meconium-stained amniotic fluid is associated with puerperal infections.. Am J Obstet Gynecol.

[30] Purushothaman SS, Park HS, Cleary PP (2004). Promotion of fibronectin independent invasion by C5a peptidase into epithelial cells in group A *Streptococcus*.. Indian J Med Res.

[31] Stalhammar-Carlemalm M, Areschoug T, Larsson C, Lindahl G (1999). The R28 protein of *Streptococcus pyogenes* is related to several group B streptococcal surface proteins, confers protective immunity and promotes binding to human epithelial cells.. Mol Microbiol.

[32] Nair S, Poyart C, Beretti JL, Veiga-Fernandes H, Berche P (2003). Role of the *Streptococcus agalactiae* ClpP serine protease in heat-induced stress defence and growth arrest.. Microbiology.

[33] Robertson GT, Ng WL, Foley J, Gilmour R, Winkler ME (2002). Global transcriptional analysis of clpP mutations of type 2 *Streptococcus pneumoniae* and their effects on physiology and virulence.. J Bacteriol.

[34] Giard JC, Rince A, Capiaux H, Auffray Y, Hartke A (2000). Inactivation of the stress- and starvation-inducible gls24 operon has a pleiotrophic effect on cell morphology, stress sensitivity, and gene expression in *Enterococcus faecalis*.. J Bacteriol.

[35] Malke H, Steiner K, McShan WM, Ferretti JJ (2006). Linking the nutritional status of *Streptococcus pyogenes* to alteration of transcriptional gene expression: the action of CodY and RelA.. Int J Med Microbiol.

[36] Kreikemeyer B, Beckert S, Braun-Kiewnick A, Podbielski A (2002). Group A streptococcal RofA-type global regulators exhibit a strain-specific genomic presence and regulation pattern.. Microbiology.

[37] Beres SB, Richter EW, Nagiec MJ, Sumby P, Porcella SF (2006). Molecular genetic anatomy of inter- and intraserotype variation in the human bacterial pathogen group A Streptococcus.. Proc Natl Acad Sci U S A.

[38] Olsen RJ, Sitkiewicz I, Ayeras AA, Gonulal VE, Cantu C (2010). Decreased necrotizing fasciitis capacity caused by a single nucleotide mutation that alters a multiple gene virulence axis.. Proc Natl Acad Sci U S A.

[39] Kreikemeyer B, Boyle MD, Buttaro BA, Heinemann M, Podbielski A (2001). Group A streptococcal growth phase-associated virulence factor regulation by a novel operon (Fas) with homologies to two-component-type regulators requires a small RNA molecule.. Mol Microbiol.

[40] Steiner K, Malke H (2002). Dual control of streptokinase and streptolysin S production by the covRS and fasCAX two-component regulators in *Streptococcus dysgalactiae subsp. equisimilis*.. Infect Immun.

[41] Shelburne SA, Keith DB, Davenport MT, Horstmann N, Brennan RG (2008). Molecular characterization of group A *Streptococcus* maltodextrin catabolism and its role in pharyngitis.. Mol Microbiol.

[42] Loughman JA, Caparon MG (2006). A novel adaptation of aldolase regulates virulence in *Streptococcus pyogenes*.. EMBO J.

[43] Pierce WA, White AG (1955). Arginine and glucose metabolism in a strain of *Streptococcus pyogenes*.. J Bacteriol.

[44] Degnan BA, Fontaine MC, Doebereiner AH, Lee JJ, Mastroeni P (2000). Characterization of an isogenic mutant of *Streptococcus pyogenes* Manfredo lacking the ability to make streptococcal acid glycoprotein.. Infect Immun.

[45] Quivey RG, Kuhnert WL, Hahn K (2001). Genetics of acid adaptation in oral streptococci.. Crit Rev Oral Biol Med.

[46] Degnan BA, Palmer JM, Robson T, Jones CE, Fischer M (1998). Inhibition of human peripheral blood mononuclear cell proliferation by *Streptococcus pyogenes* cell extract is associated with arginine deiminase activity.. Infect Immun.

[47] Lei B, Mackie S, Lukomski S, Musser JM (2000). Identification and immunogenicity of group A *Streptococcus* culture supernatant proteins.. Infect Immun.

[48] Samant S, Lee H, Ghassemi M, Chen J, Cook JL (2008). Nucleotide biosynthesis is critical for growth of bacteria in human blood.. PLoS Pathog.

[49] van de Rijn I, Kessler RE (1980). Growth characteristics of group A streptococci in a new chemically defined medium.. Infect Immun.

[50] Pettersson J, Schrumpf ME, Raffel SJ, Porcella SF, Guyard C (2007). Purine salvage pathways among *Borrelia* species.. Infect Immun.

[51] Mereghetti L, Sitkiewicz I, Green NM, Musser JM (2008). Extensive adaptive changes occur in the transcriptome of *Streptococcus agalactiae* (group B *Streptococcus*) in response to incubation with human blood.. PLoS One.

[52] Mereghetti L, Sitkiewicz I, Green NM, Musser JM (2008). Remodeling of the *Streptococcus agalactiae* transcriptome in response to growth temperature.. PLoS One.

